# Dynamical variations, impact factors, and prediction of echinoco-ccosis in Xinjiang by ARIMA-Random Forest Hybrid Model

**DOI:** 10.1371/journal.pone.0326433

**Published:** 2025-08-19

**Authors:** Fenghan Wang, Xuedong Yang, Qianqian Zhang, Zengyun Hu, Xian Zhang, Jiangshan Zhao, Nazrullozoda Sulaimon

**Affiliations:** 1 Shanghai 411 Hospital, China RongTong Medical Healthcare Group Co.Ltd./411 Hospital, Shanghai University, Shanghai, China; 2 School of Global Health, Chinese Center for Tropical Diseases Research, Shanghai Jiao Tong University School of Medicine, Shanghai, China; 3 School of Public Health, Zhengzhou University, Zhengzhou, China; 4 Center for Disease Control and Prevention of Xinjiang Uygur Autonomous Region, Urumqi, China; 5 Institute of Veterinary Medicine of the Tajik Academy of Agricultural Sciences, Dushanbe, Republic of Tajikistan; Beni Suef University Faculty of Veterinary Medicine, EGYPT

## Abstract

**Background:**

Xinjiang is the second largest pastoral areas, and the main arid and semi-arid regions in China. The echinococcosis in Xinjiang has been brought serious challenge and large pressure for the disease control and prevention.

**Methods:**

We comprehensively investigated the echinococcosis temporal variations at multiple time scales in Xinjiang during the period of 2004–2020. The relationships between the echinococcosis and the impact factors (i.e., Tmp: temperature, Pre: precipitation, RH: relative humidity, and SD: sunshine duration), and MR (medicine rate accounting in gross domestic product) are detected. Moreover, the echinococcosis is predicted by the combined model: ARIMA (autoregressive integrated moving average) and RF(random forest) hybrid model using the five factors.

**Results:**

The results indicate the echinococcosis has a significant increased trend for both confirmed cases and incidence rates with the annual trend values of 94.48 cases per year, and 0.339 new cases per 100,000 population per year. Moreover, the echinococcosis in Xinjiang has the nonlinear characteristics with the multiple periods of the 3-, 6-, 13-, 40-, and 67-month for the confirmed cases, and 3-, 6-, 12-, 34-, and 73-month for the incidence rates. In terms of the impact factors, Tmp has the positive impacts on echinococcosis, and SD has the negative impact at annual and seasonal scales. Pre has the positive impact on echinococcosis at annual, June, July and August (JJA), and September, October, and November (SON). RH has the positive relationship at JJA. MR has the significant positive relationship with echinococcosis. The ARIMA-RF hybrid model has high performance in predicting the echinococcosis variations.

**Conclusions:**

Echinococcosis in Xinjiang has a significant increased trend during the period of 2004–2020. Tmp and MR have the positive impact on the echinococcosis. The ARIMA-RF hybrid model can well predict the disease variations. Our finding can provide more characteristics about the echinococcosis variations in Xinjiang, which is the basic and important information for the disease control and prevision.

## 1. Introduction

With the ongoing global climate change and intensified human activities, the zoonotic spillover risk has been seriously increased [1]. It is found that 58% of known human pathogenic diseases have been aggravated by climate change [2]. Zoonoses seriously threaten the public health and global security and have caused the majority of recent global pandemics in humans [3]. China and the southeast Asia have the high population density and large wildlife. Their ecosystem environment is seriously impacted by the climate change and human activities. Therefore, China and the southeast Asia are the zoonosis hotspot regions [4]. Recent studies display that the living environment and behavioral patterns of the wildlife have been significantly impacted by the climate change and human activities which will cause the new contact between the humans and wildlife and eventually provide the advantages for the new epidemics of the zoonoses [1].

As one of the global distributed zoonoses prevailing in human and animals, echinococcosis is caused by adult or larval stages of tapeworms (cestodes) belonging to the genus Echinococcus (family Taeniidae) [5]. It can be found on all continents, with highest prevalence in parts of Eurasia (especially Mediterranean countries, the Russian Federation and adjacent independent states, and China), north and east Africa, Australia, and South America [6]. The alveolar echinococcosis (AE) and cystic echinococcosis (CE) are listed as the neglected tropical diseases and neglected zoonoses, which have 2–3 million people affected and 200,000 new cases diagnosed annually, and CE has more than 1 million disability-adjusted of life years (DALYs) [6–8]. AE and CE are highlighted as the second and third most important foodborne parasitic diseases by WHO and the Food and Agriculture Organization of the UN, respectively [9]. Moreover, costs of treatment for humans and economic losses to the livestock industry have been estimated larger than 2 billion dollars [10]. Only the annual global cost of CE is more than 750 million dollars, which highly exacerbates the economic burden of already low-income regions [11].

To control and decrease the CE prevalence, large efficient measures are employed. For example, the health education can effectively reduce the transmission of echinococcosis [12]. Improved control of stray dogs, echinococcidal treatments of working sheep dogs, and providing means for safe disposal of slaughtered sheep offal can lead to a decline in prevalence of E. granulosus in dogs [13]. In 2006, the Chinese government employed the national control program for echinococcosis, and the human echinococcosis prevalence had decreased from 1.08% in 2004, to 0.28% in 2016 after its completion [14–16].

The living environment of wild animals are largely controlled by the climate changes and land use and land cover which results in the echinococcus disease control depending on them [17]. Temperature (Tmp), precipitation (Pre), and relative humidity (RH) are the main climate risk factors for echinococcosis, and high RH can result in high echinococcosis risk [18]. Land surface Tmp in spring has negative impact on the prevalence of human CE in western China [19]. Echinococcus granulosus eggs are sensitive to changes in Tmp and humidity, which make them more likely to survive in low Tmp and high humidity environments [20]. The annual average precipitation has the significant nonlinear relationship with the prevalence of CE [21]. Economic condition also plays the key role on the prevalence of CE, better economy with lower prevalence [21,22].

Hence, it is important to explore the echinococcosis characteristics and the relationships related to the climate changes. As one of the high prevalence echinococcosis regions in China, Xinjiang faces the large disease control and prevention pressure because of its scarce medical resources due to the low economic level. To have an efficient control and prevention for echinococcosis in Xinjiang, three questions should be urgently answered as following (1) what are the variation characteristics of the echinococcosis? (2) what are the relationships between the echinococcosis and impact factors (climate factors and medical resource)? (3) whether the echinococcosis variations can be predicted by the impact factors using the artificial intelligence approaches?

Therefore, to address the above questions, we will explore the multiple time scale characteristics of the echinococcosis in Xinjiang during the period of 2004–2020, and detect the relationships between the echinococcosis and impact factors.

## 2. Study area, datasets, and methods

### 2.1 Study area and datasets

As the core area of the Silk Road, Xinjiang is located at the northwestern China and far from the sea covering the area of 1.66 × 10^6^ km^2^ (Fig S1 in S1 File). As controlled by the westerly circulation, Xinjiang has the arid and semiarid climate characteristics with the annual total precipitation of 150 mm and annual mean air temperature of 8°C [23]. As the second largest grazing land in China, the grassland area is 5.73 × 10^7^ hm^2^ accounting for 36% of the whole Xinjiang area. Until the end of 2022, the total number of the livestock is more than 59.85 million. As the major zoonotic in Xinjiang, the echinococcosis seriously threatens the health of the livestock and the residents.

The monthly echinococcosis data of Xinjiang is the national surveillance data downloaded from the Public Health Science Data Center of China CDC with the period of 2004–2020 (https://www.phsciencedata.cn/Share/), including the two variables of the echinococcosis confirmed case (EC) and incidence rate (IR). The climate factors include the temperature (Tmp), precipitation (Pre), relative humidity (RH), and sunshine duration (SD) which are downloaded from the China Meteorological Administration (https://data.cma.cn/). The medicine rate of the total health expenses on the GDP (Gross Domestic Product) (MR) is from the National Bureau of Statistics (https://www.stats.gov.cn/sj/ndsj/).

### 2.2 Methods

The temporal variations of the echinococcosis in Xinjiang are illustrated at annual and seasonal time scales. The four seasons are defined as spring (MAM: March, April, and May), summer (JJA: June, July, and August), fall (SON: September, October, and November) and winter (DJF: December, January, and February). The annual data and seasonal data are summed by the monthly data. The linear trend of the echinococcosis is obtained by the linear least square method and its significance is detected by the Student’s t-test at the 95% confidence level (P < 0.05).

In this study, the Ensemble empirical mode decomposition (EEMD) is used to explore the multiple periods of the echinococcosis. ARIMA model and Random Forest model are used to predict the echinococcosis variations, and the statistical metrics are used to measure the model’s performance. 80% data is used to train the model, and the other 20% data is used to test the model.

**Fig 1:**
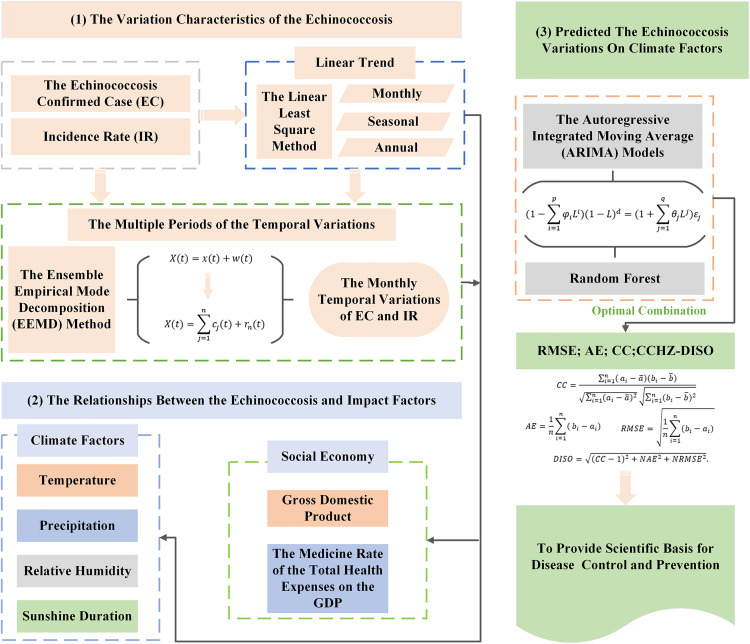
The research framework of this study.

The research framework of this study is illustrated in Fig 1.

#### 2.2.1 Ensemble empirical mode decomposition (EEMD) method.

The multiple periods of the temporal variations are analyzed by the ensemble empirical mode decomposition (EEMD) method. EEMD can well extract the multi-periods characteristics of the original time series (including the nonlinear and non-stationary time series. Because of its effectiveness, EEMD has been widely used in large areas [24]. The EEMD method is composed by the following processes. For a time series x(t), a white noise w(t) with finite amplitude is added, then we have


X(t)=x(t)+w(t) (1)

X(t) is decomposed by the intrinsic mode functions (IMFs), $c_{j}$, as


$$X(t)=\sum_{j=1}^{n}c_{j}(t)+r_{n}(t)$$ (2)

Where $r_{n}$ is the residue of X(t), after n number of IMFs with different periods are extracted. The nonlinear trend is reflected by the residue term r. After a large amount of decompositions with different noise realizations added and an ensemble, the noise added cancel each other and obtain the final oscillation components ($c_{1},c_{2},\ldots$) and the residual.

#### 2.2.2 ARIMA model and Random Forest model.

Constructed by Box and Jenkins, the Autoregressive Integrated Moving Average (ARIMA) models are one of the widely used time series models in the simulation and prediction. The basic form of ARIMA model is ARIMA (p, d, q), where the non-negative integers p and q are the orders of autoregressive and moving average polynomials respectively; d is the non-seasonal differencing required to make data stationary. An ARIMA (p, d, q) model can be expressed using lag polynomial L as the following equation


$$(1-\sum_{i=1}^{p}\varphi_{i}L^{i})(1-L{)}^{d}=(1+\sum_{j=1}^{q}\theta_{j}L^{j})\varepsilon_{j}$$ (3)

where $\varepsilon_{j}$ is a random error at time j, $\varphi_{i}$ and $\theta_{j}$ are the coefficients.

Generally, ARIMA model can capture both nonseasonal and seasonal patterns of time series. There are three steps to forecast the time series: model identification, parameter estimation, and diagnostic checking of the model. In the first step of model identification, the stationarity and seasonality of the time series are determined, which need to be modeled before parameter estimation. The augmented Dickey-Fuller (ADF) test is used to detect whether the time series is stationary. If the P values of the ADF test is less than 0.05, which indicates that the time series is stationary. If the time series is non-stationary, an autocorrelation function (ACF) plot is used to judge it as stationarity with the differencing transformation, and the parameter d is determined. Seasonality can be obtained by taking seasonal differencing and regenerating ACF and partial autocorrelation function (PACF) plots.

For the ARIMA model identification, ACF and PACF plots are also helpful to determine the values of parameters of p and q. The commonly used method: maximum likelihood is employed to estimate the parameters of the appropriately selected model. In the end, the overall adequacy of the model is checked by the Ljung and Box test. In this study, the R 4.0.5 version is applied to construct the ARIMA model in simulating and predicting the echinococcosis time series including the confirmed case time series and the incidence rate time series during the period of 2004–2020. The parameters of p and q are set as 5 and 0 based on the AIC criteria.

The fundamental concept of random forest model involves creating multiple independent decision tree models by randomly sampling data from the dataset. By averaging their prediction results, this technique mitigates errors and overfitting issues inherent in single models, thus improving the accuracy and robustness of predictions. In epidemiology, the supervised machine learning method of random forest regression has been used to predict the recent spatiotemporal spread of COVID-19 globally, yielding promising results. Studies have demonstrated that random forest outperforms other algorithms in predicting the relationships between cases, deaths, and infections of diseases such as influenza and dengue, as well as their transmission in relation to climatic factors, landscape elements, and human behaviors.

In this study, the echinococcosis is predicted by the ARIMA model and the random forest model, respectively. Then, it is predicted by the combined model of ARIMA-RF hybrid model. In the combined model, the echinococcosis is firstly predicted by the ARIMA model, and then the residual time series is predicted by the combined model.

#### 2.2.3 Statistical metrics in measuring the model’s performance.

The relationship between the echinococcosis and climate factors is measured by the correlation coefficient (CC) at 95% confidence level. After obtained the relationships, the echinococcosis variations are predicted by four artificial intelligence models.

To quantify the simulation performance of the model, some statistical metrics were employed, including the correlation coefficient (CC), absolute error (AE), root mean square error (RMSE), and Distance between Indices of Simulation and Observation (DISO) [25–27]. They are expressed as follows:


$$CC=\frac{\sum_{i=1}^{n}(a_{i}-\overset{―}{a})(b_{i}-\overset{―}{b})}{\sqrt{\sum_{i=1}^{n}{\left(a_{i}-\overset{―}{a}\right)}^{2}}\sqrt{\sum_{i=1}^{n}{\left(b_{i}-\overset{―}{b}\right)}^{2}}}$$ (4)

$$AE=\frac{1}{n}\sum_{i=1}^{n}(b_{i}-a_{i})$$ (5)

$$RMSE=\sqrt{\frac{1}{n}\sum_{i=1}^{n}\left(b_{i}-a_{i}\right)}$$ (6)

$$DISO=\sqrt{(CC-1{)}^{2}+NAE^{2}+NRMSE^{2}}$$ (7)

where *a*_*i*_ and *b*_*i*_(**i* *= 1*,* 2*, …, n*) represent the observed and simulated data, respectively. *NAE* and *NRMSE* are normalized by the average values of the observed time series.

## 3. Result

### 3.1 Linear temporal characteristics of the echinococcosis in Xinjiang at multiple time scales

**Fig 2:**
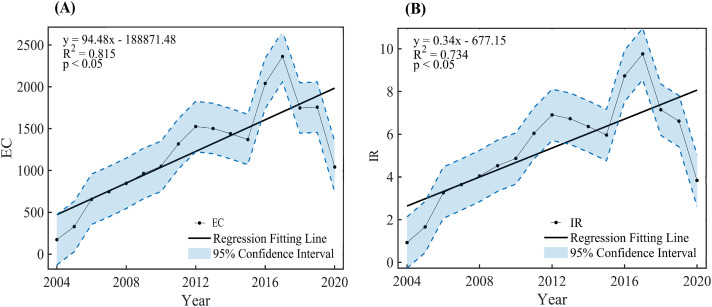
Temporal variations of the annual echinococcosis, (A) for EC (confirmed cases), and (B) for IR (cases per 100,000 population) during the period of 2004-2020, where the black straight line is the linear trend obtained by the linear least square method, the blue area is the 95% confidence interval.

In this section, the comprehensive temporal characteristics of the echinococcosis in Xinjiang at different time scales are displayed in Fig 2, including the annual, seasonal and monthly scales. For the annual echinococcosis, the average value of EC is 1288 during the period of 2004–2020 with the maximum value of 2362 in 2017, and the minimum value of 173 in 2004, and the average value of IR is 5.35% with the corresponding maximum value of 9.76%, and the minimum value of 0.93% (Fig 2, Table S1 in S1 File). A significant increasing linear trend (P < 0.05) of EC is observed with the value of 94.79 per year in 2004–2020. Particularly, the annual echinococcosis is persistently increased from 173 in 2004–1525 in 2012, and decreased in 2013–2015 with the values of 1502, 1440, and 1370, respectively. It becomes increased tendency in 2016 and 2017 with the values of 1915 and 2488. And then, the confirmed number is decreased at 2018, 2019, and 2020 with the values of 1747, 1757, and 1046 (Fig 2A). For IR, it has the significant increasing linear trend with the value of 0.34% per year (P < 0.05), and the temporal variation is similar as the EC (Fig 2B). The largest value in 2017 mainly is caused by the mass screening, and the decreased values of echinococcosis after 2017 is resulted from the efficient control measurements.

MAM, JJA, SON, and DJF have the significant linear increased tendencies with the values of 24.32, 23.93, 20.19, and 25.95 per year for EC, and 0.09%, 0.08%, 0.06%, and 0.13% per year for IR (Fig S2 in S1 File), For the intra-annual variation, it is decreased from January to October, and it becomes increase to December(Fig S3 in S1 File). The monthly EC and IR have the significant positive linear trends with the values of 0.648 per month, and 0.002 per month during the (Fig S4 in S1 File, Text S1).

### 3.2 Multiple periods of the echinococcosis variations obtained by EEMD

**Fig 3:**
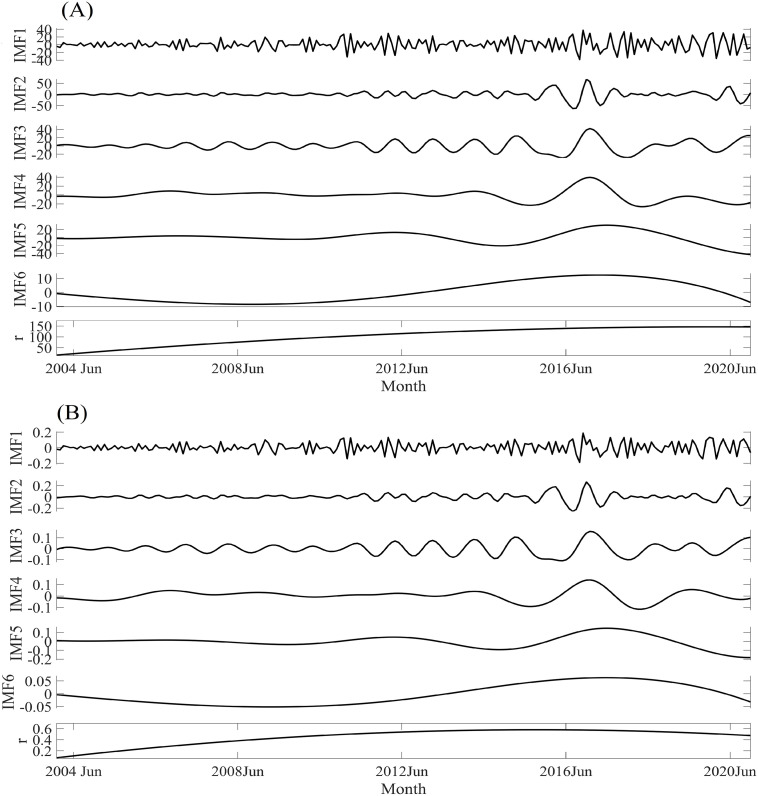
The decomposition results of the monthly confirmed cases (A) and incidence rates (B) in Xinjiang obtained by EEMD during the period of 2004-2020, where IMF1, IMF2, IMF3, IMF4, IMF5, and IMF 6 are the decomposed period time series, and r is the nonlinear trend.

The multiple periods of the confirmed cases and incidence rates of the echinococcosis in Xinjiang during the period of 2004–2020 are decomposed by EEMD in Fig 3. It shows that the confirmed cases have multiple periods with the values of 3, 6, 13, 40, 67, and 190 months for IMF1, IMF2, IMF3, IMF4, IMF5 and IMF6, and the corresponding contributions are 21%, 23%, 16%, 14%, 21%, and 5% (Fig 3A, Table S2 in S1 File). For the incidence rates, the EEMD results are similar as the results of the confirmed cases. The multiple periods of the incidence rate are 3, 6, 12, 34, 73, and 190 months for IMF1, IMF2, IMF3, IMF4, IMF5 and IMF6 with the corresponding contributions of 22%, 20%, 14%, 11%, 24%, and 8% (Fig 3B, Table S2 in S1 File).

The above results suggest that the echinococcosis variations in Xinjiang during the period of 2004–2020 have multiple periods, which can be used for the prediction in future and provide important information for the disease control and prevision.

### 3.3 Relationships between the echinococcosis and the impact factors in Xinjiang during the period of 2004–2020

**Fig 4:**
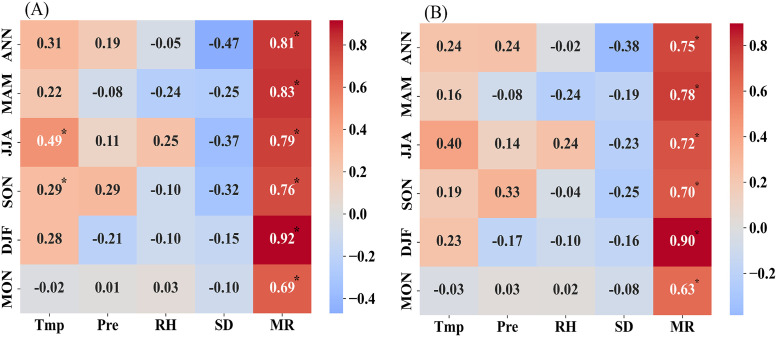
Correlation coefficient results between the echinococcosis and the impact factors during the period of 2004-2020 at multiple time scales: ANN, MAM, JJA, SON, DJF, and MON, where (A) for EC, (B) for IR.

The temporal characteristics of the impact factors at multiple time scales are provided in the Text S2, which show the significant positive trend of Tmp with the value of 0.03 °C per year, RH (−0.17 per year), and SD (−8.53 per year) (Table S3 in S1 File). The relationships between the echinococcosis and the impact factors in Xinjiang are explored during the period of 2004−2020. The CC values are applied to measure their relationships in Fig 4, and the significant is detected by the t test at 95% confidence level. For the annual scale, the MR has the largest positive impact on the echinococcosis among the five factors, and the CC values are 0.81 for EC, and 0.75 for IR (Fig 4). Followed by the Tmp and Pre with the positive impacts on the echinococcosis, and the corresponding CC values are 0.31 and 0.19 for EC, and 0.24 and 0.24 for IR. SD has the negative impact on EC and IR with the CC values of −0.47 and −0.38.

For the seasonal scale, Tmp has the positive impact on EC and IR at the four seasons with the largest impact at JJA (CC = 0.49, 0.4), and followed at SON, DJF, and MAM. Pre has the positive impact on EC and IR at JJA (CC = 0.11, 0.14) and SON (CC = 0.29, 0.33), It is negative correlation between Pre and echinococcosis at DJF with the CC values of −0.21 for EC and −0.17 for IR. For RH, except the positive correlation with echinococcosis (CC = 0.25 for EC, 0.24 for IR), it has the negative impact on echinococcosis at the other three seasons, and the strongest negative impact appears at MAM with the CC = −0.24 for both EC and IR. SD has the negative impact on echinococcosis at all the seasons with the largest magnitudes at JJA. For MR, it has the largest significant positive impact (P < 0.05) on echinococcosis than the other factors in all the seasons, with the CC values of 0.83, 0.79, 0.76, and 0.92 for EC, 0.78, 0.72, 0.7, 0.9 for IR. For the monthly scale, MR has the significant positive impact on echinococcosis, and the other factors have no significant impact (Fig 4).

### 3.4 Prediction of the echinococcosis by ARIMA-RF hybrid forecast model

**Fig 5:**
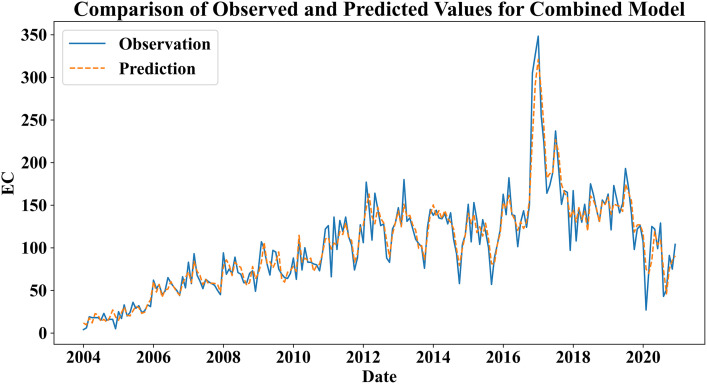
Prediction results of the echinococcosis confirmed cases obtained by the combined model: ARIMA-RF hybrid model.

Based on the above result in Section 4, in this section, we provide the prediction results compared by the single model (i.e., ARIMA and RF) and combined model (ARIMA-RF Hybrid), which illustrate that the echinococcosis variations of Xinjiang will be well predicted by ARIMA-RF hybrid model. The prediction results are displayed in Figs S5 and S6 in S1 File, and Fig 5, and the models’ performances are evaluated by CC, AE, RMSE, and DISO in Table S4 in S1 File. Because the echinococcosis confirmed cases and the incidence rates have the similar temporal variations, the echinococcosis confirmed cases is only predicted by the above three models.

Fig S5 in S1 File shows that the temporal variations of the echinococcosis confirmed cases are well captured by the ARIMA model. Moreover, the peak values are also predicted. The corresponding statistical metrics are 19.48, 27.77, 0.87, and 1.42 for AE, RMSE, CC, and DISO, respectively (Table S4 in S1 File).

The ARIMA prediction is only based on the temporal characteristics of the echinococcosis confirmed cases. However, the four climate factors (i.e., Tmp, Pre, RH, and SD) and MR are not considered in the model. Therefore, the four climate factors and MR are included as the input data to predict the echinococcosis confirmed cases by RF. The prediction result of random forest model is provided in Fig S6 in S1 File.

Fig S6 in S1 File show that the prediction result of random forest considering the four climate factors and MR has the higher accurate than the result of ARIMA in Fig S5 in S1 File. The statistical metrics are 12.53, 19.31, 0.95, and 0.95 for AE, RMSE, CC, and DISO, respectively (Table S4 in S1 File). However, the prediction of the peak values should be improved. Then, the combined model of ARIMA-RF is employed to improve the model’s performance (Fig 5). In the combined model, the echinococcosis confirmed cases are first predicted by ARIMA, and the residual time series is predicted by random forest. The predicted result is displayed in Fig 5, which indicates that the prediction of the combined model has the best performance than the ARIMA and random forest. The corresponding statistical metrics of the combined model are 9.81, 13.99, 0.97, and 0.71 for AE, RMSE, CC, and DISO in Table S4 in S1 File.

## 4. Discussion

As one of the twenty neglected tropical diseases by the WHO and targeted for control for more than several decades, the echinococcosis poses a significant threat to public health and livestock industry [28,29] More than one million people are globally affected by echinococcosis, and the annual cost of cost of treatment and losses to the livestock industry is estimated to be $760 million [30]. In western China, about 50 million people are at the risk of echinococcosis infection [31]. Therefore, taking the Xinjiang as a case study, we comprehensively investigate the echinococcosis characteristics and predict its temporal variations based on the combined model: ARIMA-RF.

The linear trends of the temporal variations and the multiple periods of the echinococcosis in Xinjiang are obtained during the period of 2004–2020. The clinical symptoms and diagnosis of the echinococcosis are not analyzed. According to the ultrasonography screening of hepatic cystic echinococcosis in sheep flocks in a county of Xinjiang [32], it suggested that culled aged sheep play a key role in the transmission of CE. A deep convolutional neural network model was developed to identify echinococcosis and its types, which indicated that this model showed significantly better performance compared with senior radiologists from a high-endemicity area [33]. The epidemic characteristics of the human cystic and alveolar echinococcosis in Kyrgyzstan were explored.

Our previous studies constructed the dynamic models using the ordinary difference equations to explore the effects of increasing the sheep number and health education on the echinococcosis control [12,34,35]. This study is using the combined model of ARIMA-RF to predict the temporal variations of the echinococcosis using the four climate factors and MR, which can provide important scientific basis for the disease control and prevention. Our result suggest that RH has the positive relationship which is consistent with some previous works [18,20]. MR has the significant positive relationship with echinococcosis, which shows that the regions have better economy with lower prevalence for CE [22].

Besides the four climate factors and MR, other driving factors are also important for the CE prevalence. Health education, improvements in sanitation, and interventions targeted at humans (ultrasound screening, surgical and albendazole treatment) and dogs (management and deworming) were the main measures implemented [12,18].In order to investigate the dynamic variations of the echinococcosis transmission between impact factors, human, dogs, and livestock, more datasets should be added, such as the dog data and the livestock data, and more impact factor data. Other statistical models are also applied to predict the diseases [36–38]. A novel Bayesian spatio-temporal model is proposed to predict emerging infectious disease [37]. The multivariable linear regression model and the stepwise regression model, the multinomial logistic regression model, the naive Bayesian classification model, and the classification and regression tree model (CART) was established, and the CART model had the highest accuracy, sensitivity, and specificity values, and the multinomial logistic regression model had the highest precision value [38].Overall, more important research topics about the echinococcosis in Xinjiang should be illustrated in future. For example, the spatial distribution of the echinococcosis can be analyzed when the related data is available. The dynamic epidemic model is necessary to explore the dynamic behaviors of the echinococcosis transmission among human and animals. Moreover, the echinococcosis disease burden is also urgent.

## 5. Conclusion

In this study, we firstly investigated the echinococcosis temporal variations (linear trend) at multiple time scales: annual, seasonal and monthly scales in Xinjiang during the period of 2004–2020. The nonlinear characteristics (multiple periods) of the echinococcosis are explored by the EEMD method. The relationships between the echinococcosis and the four climate factors (i.e., Tmp, Pre, RH, and SD), and MR are also detected from the annual, seasonal, and monthly scales. At last, the echinococcosis is predicted by two single models: ARIMA and random forest, and their combined model. The major results are concluded as follows.

(1) The echinococcosis in Xinjiang has a significant increased trend for both confirmed cases and incidence rates. The annual linear trend of the confirmed cases and incidence rates are 94.48 per year, and 0.339 per year, and the corresponding monthly linear trends are 0.65 per year, and 0.0023 per year.
(2) According to the EEMD result, the echinococcosis in Xinjiang has the nonlinear characteristics with the multiple periods of the 3-, 6-, 13-, 40-, and 67-month for the confirmed cases, and 3-, 6-, 12-, 34-, and 73-month for the incidence rates.
(3) Among the climate factors, Tmp has the positive impacts on echinococcosis, and SD has the negative impact at annual and seasonal scales. Pre has the positive impact on echinococcosis at annual, JJA, and SON. RH has the positive relationship at JJA. MR has the significant positive impact with the CC values larger than 0.6, which indicates that the increased medical resource can detect more confirmed cases.
(4) ARIMA model and random forest model can capture the echinococcosis temporal variations in Xinjiang. The combined model of the ARIMA-RF has the best performance than the two single models with the CC value of 0.97, and DISO value of 0.71.

Echinococcosis is one of the important zoonoses in Xinjiang, which plays a key role for the social-economic development and the local human health. The impact factors and high accurate simulation and prediction of the echinococcosis variations can provide the basic and important information for the disease control and prevision. In our future study, we should focus on the one health concept including the environment, animal, and human. More environment data, more animal data (e.g., dog, cattle and sheep), and more confirmed case data are still the important scientific information to reveal more echinococcosis characteristics, to predict or even early warn the echinococcosis transmission. In the end, based on the more echinococcosis characteristics, higher accurate prediction, we can propose more scientific and reasonable response measures to the local government.

## Supporting information

S1 File PLOS ONE supplementary material mouthguard compliance. (DOCX)
