# Supplementary material for: Dynamical variations, impact factors, and prediction of echinoco-ccosis in Xinjiang by ARIMA-Random Forest Hybrid Model
Source: PLoS One. 2025 Aug 19;20(8):e0326433. doi: 10.1371/journal.pone.0326433 (PMC12364351; doi:10.1371/journal.pone.0326433)
Supplement: S1 File — (DOCX) [file pone.0326433.s001.docx]

Supporting Information for

**Dynamical variations, impact factors, and prediction of echinoco-ccosis in xinjiang by ARIMA-Random Forest Hybrid Model**

Fenghan Wang^1^, Xuedong Yang^1^, Qianqian Zhang^2^, Zengyun Hu^2*^, Xian Zhang^3^, Jiangshan Zhao^4^, Nazrullozoda Sulaimon^5^

^1^Shanghai 411 Hospital, China RongTong Medical Healthcare Group Co.Ltd./411 Hospital, Shanghai University, Shanghai, China

^2^School of Global Health, Chinese Center for Tropical Diseases Research, Shanghai Jiao Tong University School of Medicine, Shanghai, China

^3^School of Public Health, Zhengzhou University, Zhengzhou, China

^4^Center for Disease Control and Prevention of Xinjiang Uygur Autonomous Region, Urumqi, China

^5^Institute of Veterinary Medicine of the Tajik Academy of Agricultural Sciences. Dushanbe, Republic of Tajikistan

*****Corresponding author

E-mail: [hzyhjq@sjtu.edu.cn](mailto:hzyhjq@sjtu.edu.cn)(ZH)

Table S1, Statistical characteristics of the echinococcosis (EC and IR) in Xinjiang during the period of 2004-2020 at the annual and seasonal scales, including the mean value, maximum value and the corresponding year, minimum value, and the corresponding year.

| **Variable** | **Time scale** | **Mean** | **Max** | **Max year** | **Min** | **Min year** |
| --- | --- | --- | --- | --- | --- | --- |
| **EC** | ANN | 1228 | 2362 | 2017 | 173 | 2004 |
|  | MAM | 326 | 560 | 2017 | 55 | 2004 |
|  | JJA | 309 | 619 | 2017 | 56 | 2004 |
|  | SON | 263 | 509 | 2016 | 47 | 2004 |
|  | DJF | 333 | 1138 | 2016 | 47 | 2004 |
| **IR** | ANN | 5.35 | 9.76 | 2017 | 0.93 | 2004 |
|  | MAM | 1.44 | 2.34 | 2017 | 0.3 | 2004 |
|  | JJA | 1.36 | 2.58 | 2017 | 0.3 | 2004 |
|  | SON | 1.4 | 3.76 | 2016 | 0.24 | 2004 |
|  | DJF | 1.44 | 2.83 | 2017 | 0.08 | 2004 |

Table S2. Periods (months) and Contributions (%) of different IMF of the confirmed cases and incidence rate decomposed by EEMD during the period of 2004-2020.

|  | **Confirmed cases** | | **Incidence rate** | |
| --- | --- | --- | --- | --- |
| **IMF** | **Period** | **Contribution** | **Period** | **Contribution** |
| **IMF1** | 3 | 21 | 3 | 22 |
| **IMF2** | 6 | 23 | 6 | 20 |
| **IMF3** | 13 | 16 | 12 | 14 |
| **IMF4** | 40 | 14 | 34 | 11 |
| **IMF5** | 67 | 21 | 73 | 24 |
| **IMF6** | 190 | 5 | 190 | 8 |

Table S3. Linear trend of impact factors: Tmp (℃/year), Pre (mm/year), RH (%/year), SD (h/year), and MR (%/year) at annual and seasonal scales, where * indicates the linear trend is significant at 95% confidence level.

| **Time scale** | **Tmp** | **Pre** | **RH** | **SD** | **MR** |
| --- | --- | --- | --- | --- | --- |
| **ANN** | 0.03* | -0.36 | -0.17* | -8.53* | 0.38* |
| **MAM** | 0.07* | -0.33 | -0.24* | 1.36 | 0.09* |
| **JJA** | 0.04* | 0.31 | 0.02 | -5.04* | 0.09* |
| **SON** | -0.04 | 0.16 | -0.19* | -0.38* | 0.09* |
| **DJF** | 0.03 | -0.49* | -0.14 | -2.59 | 0.09* |

Table S4. Statistical metric results of the ARIMA, Random Forest, and ARIMA-Random Forest Hybrid Model performance.

| **Model** | **MAE** | **RMSE** | **CC** | **DISO** |
| --- | --- | --- | --- | --- |
| **ARIMA** | 19.48 | 27.77 | 0.87 | 1.42 |
| **Random Forest** | 12.53 | 19.31 | 0.95 | 0.95 |
| **ARIMA-Random Forest** | 9.81 | 13.99 | 0.97 | 0.71 |

**
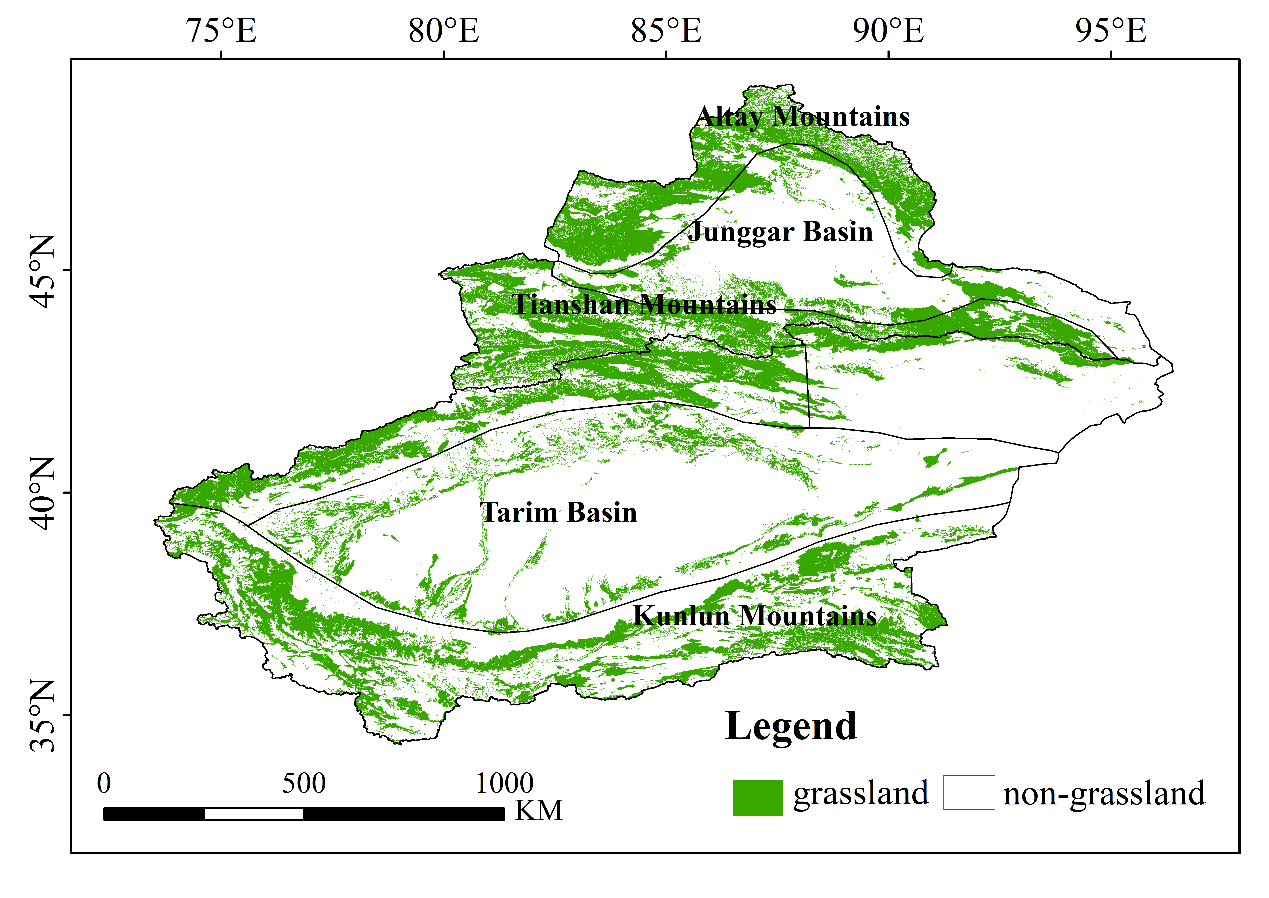
**

Fig S1. Study area of Xinjiang, including the grassland distribution.


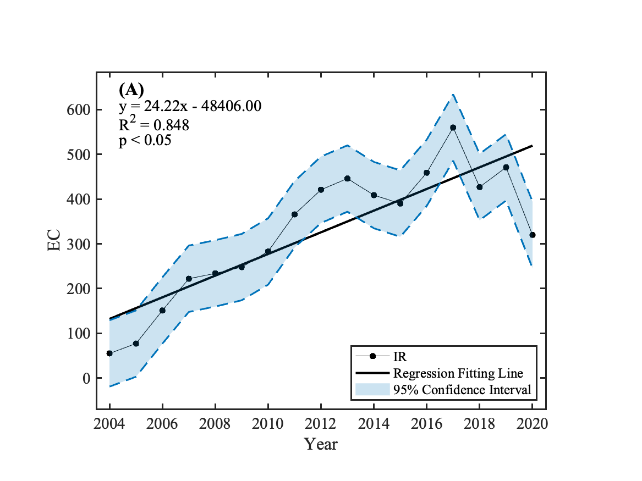

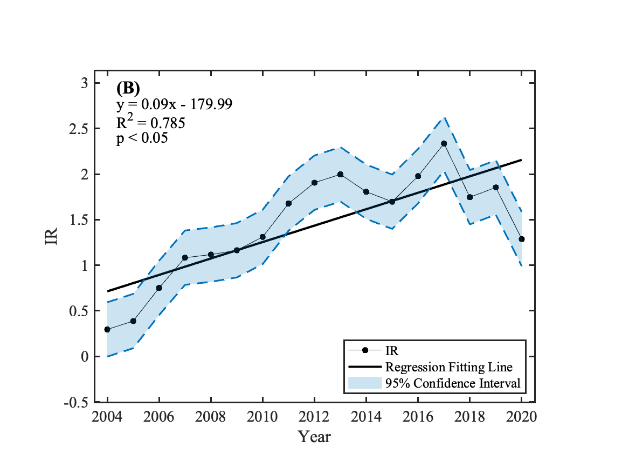


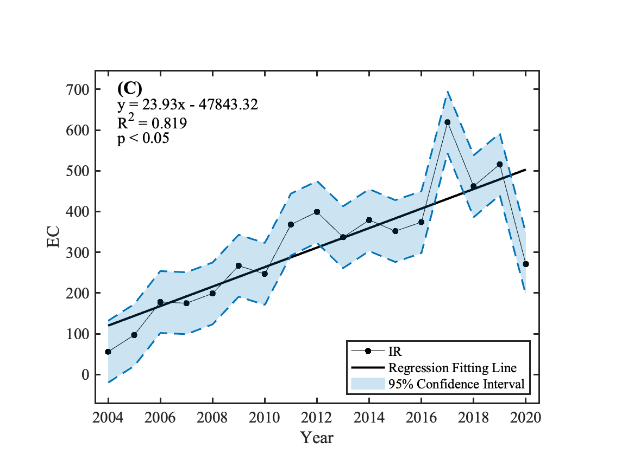

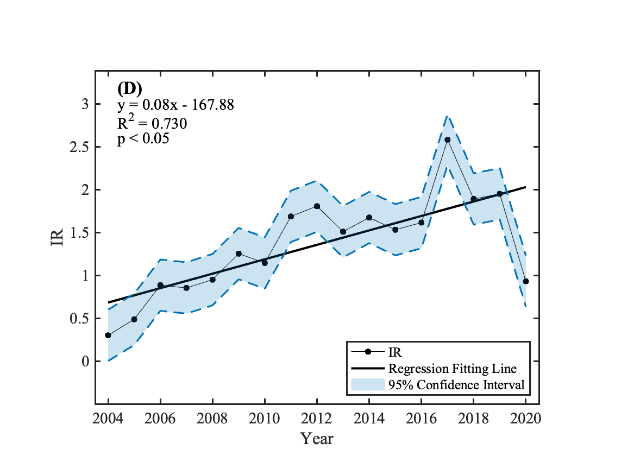


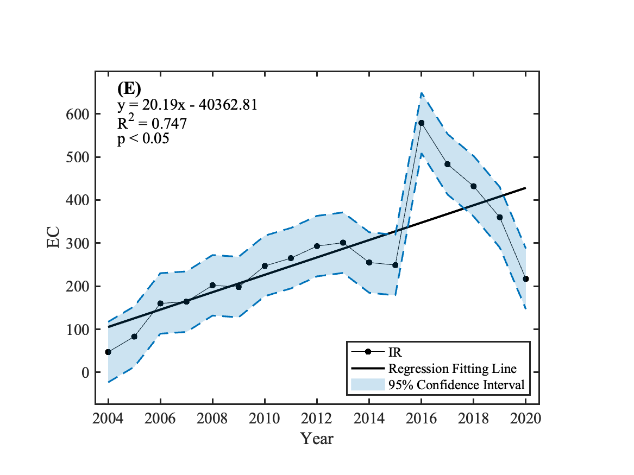

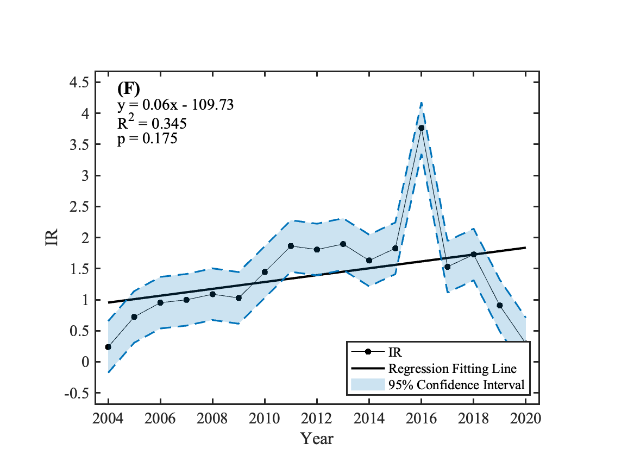


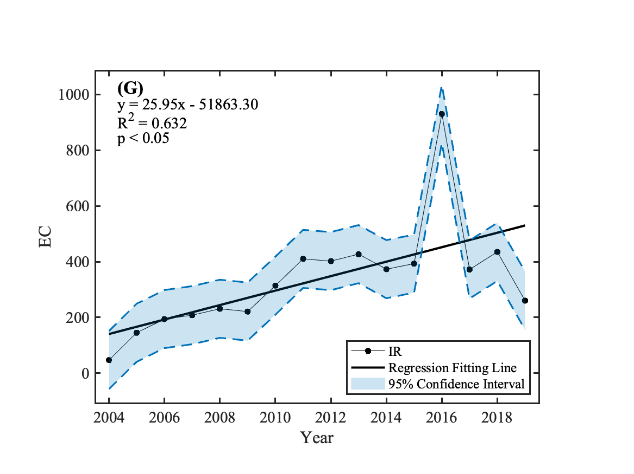

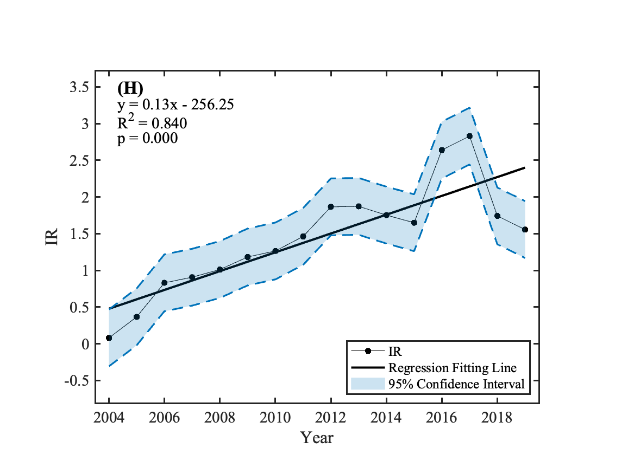


Fig S2. The seasonal temporal variations of the echinococcosis, where (A), (C), (E), and (G) for MAM, JJA, SON, and DJF of EC, and (B), (D), (F), and (H) for MAM, JJA, SON, and DJF of IR.


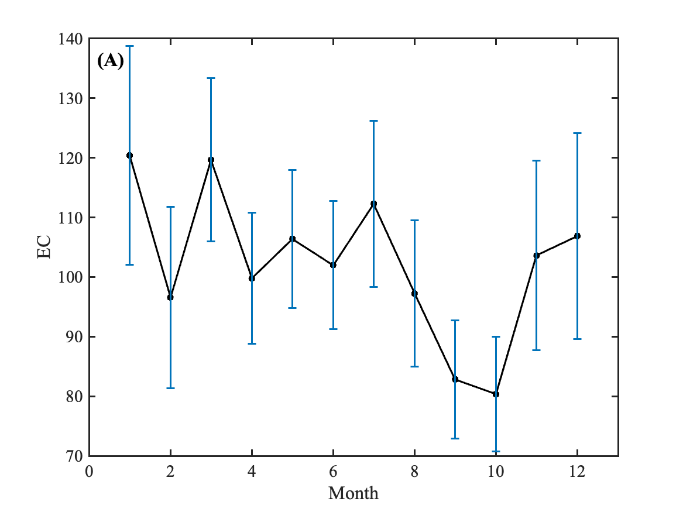

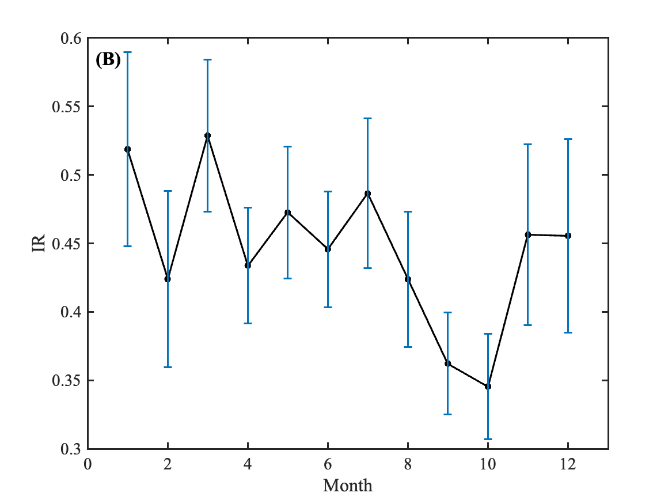


Fig S3. Intra-annual echinococcosis variations during the 2004-2020, where (A) for EC, and (B) for IR.


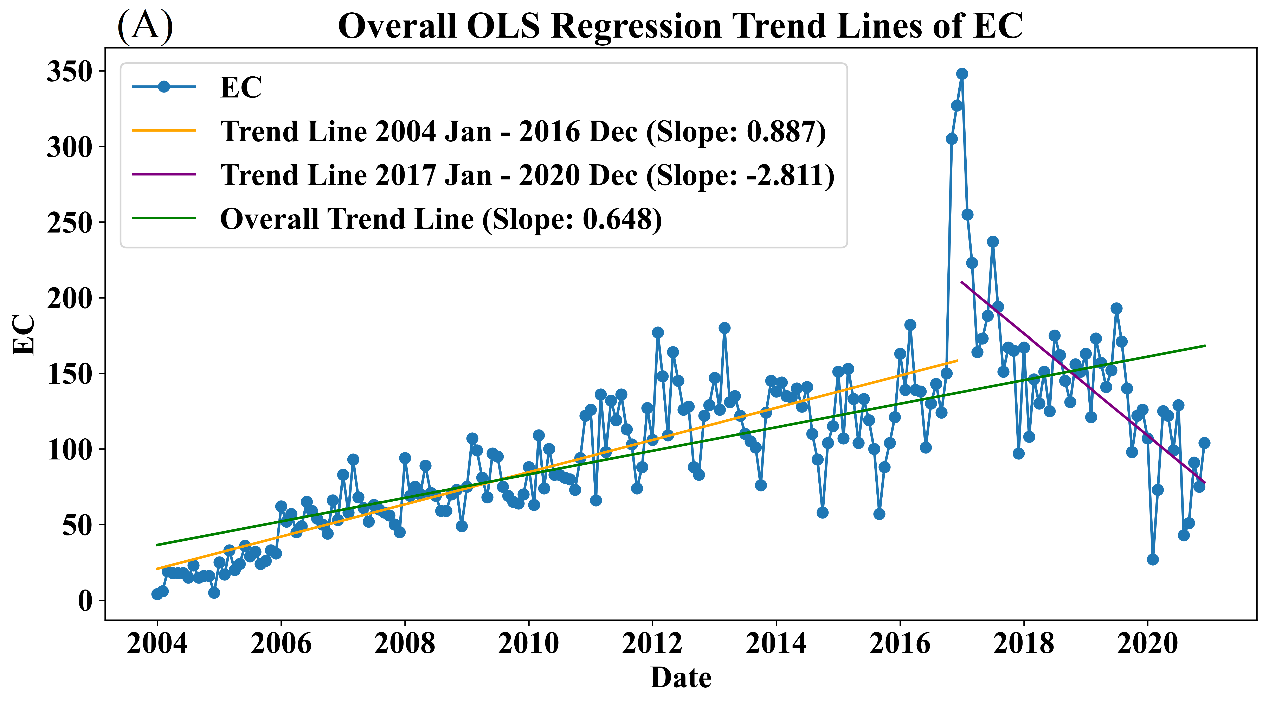

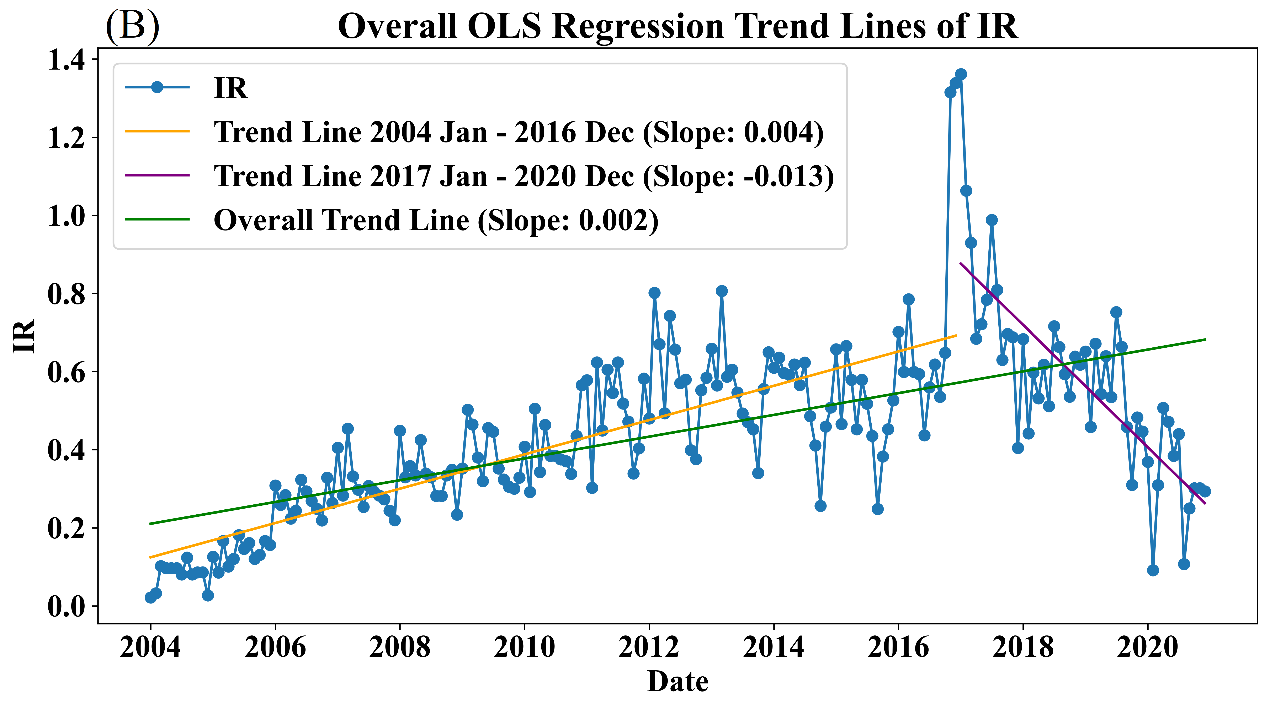


Fig S4 The monthly temporal variations of the echinococcosis for the confirmed cases (A), and incidence rates (B) during the period of 2004-2020, where the red straight line, yellow straight line, and blue straight line are obtained by the least linear regression method for the periods of 2004 Jan-2016 Dec, 2017 Jan-2020 Dec, and 2004 Jan-2020 Dec, respectively.


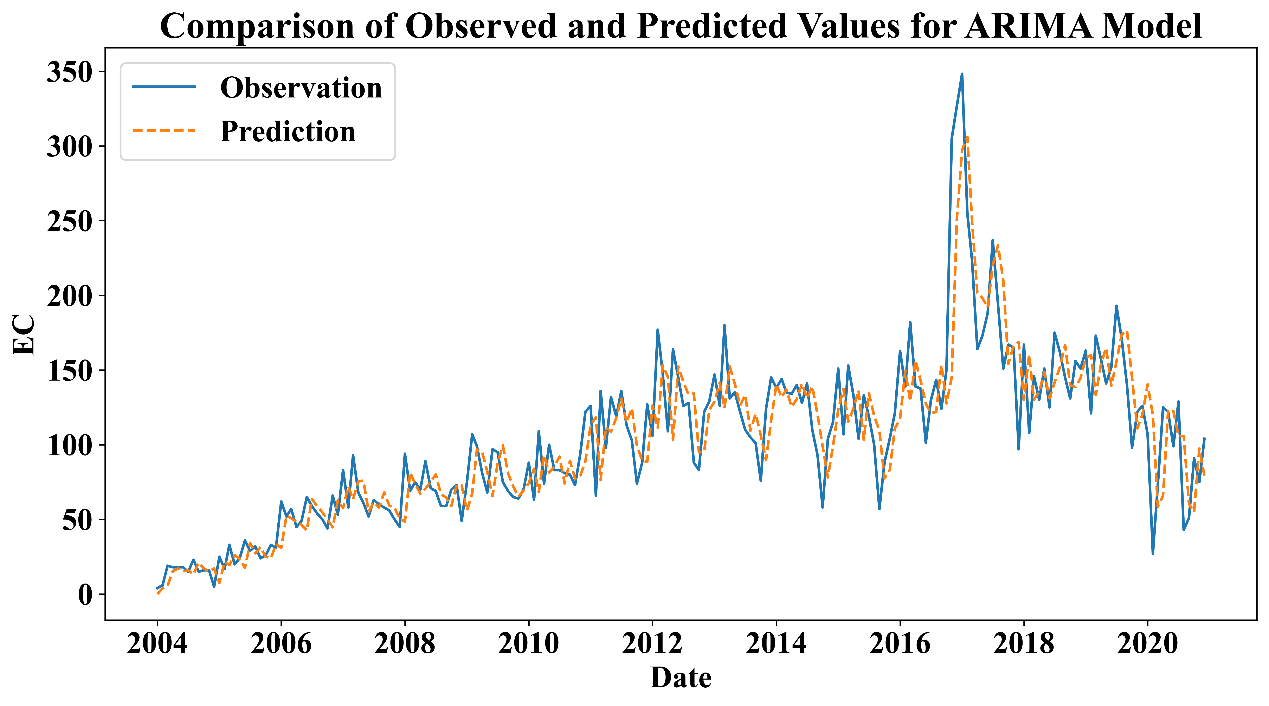


Fig S5. Prediction results of the echinococcosis confirmed cases obtained by ARIMA model.


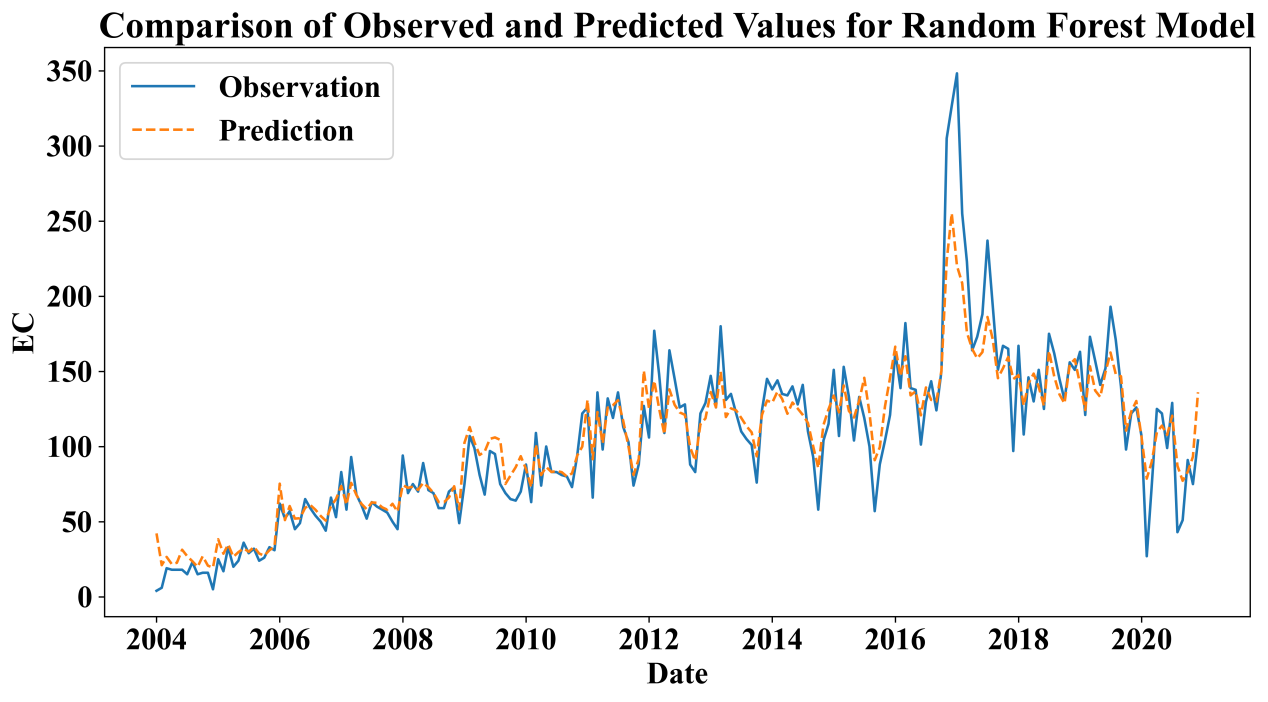


Fig S6. Prediction results of the echinococcosis confirmed cases obtained by RF model.

**Text S1 Seasonal and monthly variations of echinococcosis.**

For the seasonal variations, the averaged numbers of the EC/IR are 326/1.44%, 309/1.36%, 263/1.4% and 333/1.44% for MAM, JJA, SON, and DJF, respectively (Table S1). The corresponding minimum values of the four seasons are observed at the same year of 2004 with the values of 55/0.3%, 56/0.3%, 47/0.24%, and 47/0.08%. The maximum values of EC of MAM and JJA are 560, and 619 at the same year of 2017. For SON and DJF, they have the maximum EC at 2016 with the values of 509, and 1138. IR in MAM, JJA, and DJF has the maximum values (2.34%, 2.58%, and 2.83%) at the same year of 2017, and in SON the maximum value is 3.76% at 2016. In terms of the linear trend results, the four seasons of MAM, JJA, SON, and DJF have the significant linear increased tendencies with the values of 24.32, 23.93, 20.19, and 25.95 per year for EC, and 0.09%, 0.08%, 0.06%, and 0.13% per year for IR (Figure S2).

For the averaged monthly confirmed cases in 2004-2020, January has the maximum confirmed cases with the value of 127, and October has the minimum confirmed case with the value of 79 (Figure S3A). In fact, the confirmed cases have the decreased tendency from January to October.

For the monthly echinococcosis, the EC and IR have the significant positive linear trends with the values of 0.648 per month, and 0.002 per month during the (Figure S4). It shows that the echinococcosis confirmed cases, and incidence rates have the significant increasing trend from 2004 Jan to 2016 Dec (0.887 per month, and 0.004 per month), and then significantly decrease from 2017 Jan to 2020 Dec with the values of (-2.811 per month, and -0.013 per month).

**Text S2 Temporal characteristics of the impact factors at multiple time scales**

Echinococcosis variations are impacted by some factors, which can change the echinococcosis transmission characteristics. Therefore, in this section, we detect the temporal characteristics of the impact factors by the linear least square method at annual, seasonal and monthly scales during the period of 2004-2020. The impact factors include the Tmp, Pre, RH, SD, and MR.

During 2004-2020, the annual Tmp has a significant positive linear trend with the value of 0.03 ℃/year (p<0.05) (Table S3). A weak negative linear trend (-0.36 mm/year) is observed for the annual Pre. RH and SD have the significant negative linear trends with the values of -0.17/year, -8.53 h/year (p<0.05). For MR, it has the significant increasing trend with the value of 0.38%/year, which shows that the medical resource investigation in Xinjiang has great increase to protect the public health.

For the seasonal variations, Tmp and MR have significant positive linear trends (k=0.07 ℃/year, k=0.09%/year, p<0.05) in MAM. A significant negative linear trend with the value of -0.24/year for RH is observed in MAM. The precipitation and SD in MAM have the negative linear trends with the values of -0.33 mm/year, and -1.36 h/year. For JJA, the significant positive linear trends are observed for Tmp and MR with the values of 0.04 ℃/year and 0.09%/year (p<0.05), and SD has the significant negative linear trend with the value of 0.02/year (p<0.05). Pre and RH have the temporal fluctuation with no significant linear tendency (Table S3).

Tmp, RH, and SD of SON have the significant negative linear trends with the values of -0.04 ℃/year, -0.19/year, and -3.08 h/year, respectively, and MR also has the significant positive linear trend in SON same as the values in MAM and JJA. For DJF, only Pre has a significant negative linear trend with the value of -0.49 mm/year (p<0.05), and the linear trends of the other climate factors are not significant at 95% confidence level (Table S3). The positive linear trend of MR has the value of 0.09%/year significantly at the 95% confidence level.

**References:**

Hu, Z., Chen, D., Chen, X., et al., 2022, CCHZ-DISO: A Timely New Assessment System for data quality or model performance from Da Dao Zhi Jian, Geophysical Research Letters, 49, e2022GL100681.

Hu, Z., Chen, X., and Zhou, Q., et al., 2019, DISO: A rethink of Taylor diagram, International Journal of Climatology, 39(5): 2825-2832.

Hu, Z., Zhou, Q., Chen, X., Qian, C., Wang, S., Li, J., 2017, Variations and changes of annual precipitation in Central Asia over the last century. International Journal of Climatology, 37,157-170.

Peng, Y., Zhang, H. and Hu., Z., et al., 2024, Future challenges of terrestrial water storage over the arid regions of Central Asia, International Journal of Applied Earth Observation and Geoinformation, 132, 104026.

Zhang, H., Hu, Z. and Zhang, Zhuo, et al., 2024, How does vegetation change under the warm–wet tendency across Xinjiang, China?, International Journal of Applied Earth Observation and Geoinformation, 127, 103664.

Zhou, Q., Chen, D., and Hu, Z. et al, 2021, Decompositions of Taylor diagram and DISO performance criteria, International Journal of Climatology, 41 (12), 5726-5732.
